# Preparation and Characterization of Bacterial Cellulose/Carboxymethyl Cellulose Composite Films

**DOI:** 10.3390/ma19102038

**Published:** 2026-05-13

**Authors:** Liang Wang, Wendi Li, Yunfa Lu, Sai Xu

**Affiliations:** School of Food and Bioengineering, Jiangsu University, Zhenjiang 212013, China

**Keywords:** bacterial cellulose, carboxymethyl cellulose, citric acid post-treatment, in situ composite, water management, moisture-management materials

## Abstract

**Highlights:**

- In situ CMC incorporation densified the BC nanofiber network without changing cellulose I.
- Citric acid post-treatment possibly promoted ester-linkage-assisted network stabilization and contributed to improved integrated properties.
- BC/CMC-3 favored retention, strength, and barrier performance, whereas BC/CMC-4 favored re-hydration.
- CMC dosage can be used to tailor BC films for moisture-management-related applications.
- The strategy provides a simple route for regulating water management and mechanics simultaneously.
- The XNH6-based system shows promise for high-value BC composite material development.

**Abstract:**

Bacterial cellulose (BC) is a natural nanofibrous material, but its limited functional tunability restricts its use in moisture-management applications. In this study, BC/carboxymethyl cellulose (CMC) composite films were prepared by in situ fermentation using a kombucha-derived high-yield strain, followed by citric acid post-treatment. The films were characterized by FTIR, XPS, XRD, SEM, and physicochemical tests. CMC incorporation regulated the BC nanofibrous network while preserving cellulose I, and citric acid post-treatment promoted possible ester-linkage-assisted network stabilization. The modified films showed improved water-management behavior, water-vapor barrier performance, and mechanical response while maintaining high surface hydrophilicity; notably, BC/CMC-3 reduced the water vapor transmission rate from 106.13 ± 4.24 to 71.07 ± 2.46 g/(m^2^·24 h). These results suggest that CMC dosage is an effective strategy for tailoring BC-based films for moisture-management applications.

## 1. Introduction

Bacterial cellulose (BC) is a natural nanofibrous material produced by microorganisms such as acetic acid bacteria [1,2]. Compared with plant cellulose, BC has several distinct advantages [3,4]. It has very high chemical purity and contains no lignin or hemicellulose; it also has an ultrafine three-dimensional nanofiber network, which gives it high mechanical strength and good biocompatibility [5]. These properties give BC broad application prospects in artificial skin, vascular scaffolds, flexible electronic devices, high-performance composites, and food packaging [6,7,8,9]. However, native BC also has some limitations in its physicochemical properties [10,11,12]. In particular, the lack of tunable functional groups on its molecular chains results in limited functionality [13]. In addition, under certain drying conditions or in complex physiological environments, the water-retention ability and swelling behavior of BC may not meet the requirements of high-precision applications [14].

In recent years, combining BC with different polymer materials has become an important way to improve its functionality and expand its applications [15]. Current studies mainly focused on combining BC with natural or synthetic polymers to impart additional functional characteristics [12]. For example, BC has been physically blended with poly(vinyl alcohol) or gelatin to improve mechanical performance and biocompatibility, with potential applications in drug delivery and biomedicine [16,17]. Meanwhile, with the growing demand for biocompatible materials, BC-based composites with natural polysaccharides such as sodium alginate and chitosan have attracted considerable attention [18,19,20]. Previous studies have shown that interactions between sodium alginate and BC can significantly improve the hydration behavior and mechanical properties of the resulting composite films, thus supporting their use in artificial skin and wound dressing applications [21].

However, compared with other BC composite systems, studies on BC/carboxymethyl cellulose (CMC) composites remain relatively limited, and most reports focus on application-oriented hydrogel or film systems [12,22,23]. Although CMC has been shown to improve the re-hydration, swelling, and water-absorption behavior of BC-based materials, most reported systems have been prepared by conventional physical methods such as direct impregnation, simple deposition, or blending [24]. For example, direct deposition of CMC into the BC network can produce BC/CMC hydrogels with good re-hydration capacity, and BC/CMC composite hydrogels prepared by simple immersion also show high swelling ability [25]. However, these physical methods mainly rely on hydrogen bonding, van der Waals forces, or electrostatic interactions to combine the components. Thus, systematic studies on the intermolecular interactions between BC and CMC, the interfacial binding mode, and the structure–property relationship with macroscopic performance are still lacking [26]. Previous studies also showed that simple blending of BC and CMC can improve hydration behavior, but the resulting materials are still inadequate in mechanical properties and structural stability, which limits their use in high-performance applications [27]. Therefore, developing a controllable strategy to regulate the mode of combination between BC and CMC and thereby improve the integrated properties of the composites remains a key research challenge.

In this study, BC/CMC composite films were prepared by an in situ compositing strategy using the high-yield BC-producing Novacetimonas hansenii XNH6 strain screened in our laboratory, and different concentrations of CMC (0.2, 0.4, 0.6, 0.8, and 1.0 g/L) were introduced during BC growth. To further improve structural stability, citric acid post-treatment was applied to the BC/CMC films, with the aim of promoting possible ester-linkage-assisted interactions within the nanofiber network. FTIR, XPS, SEM, and XRD were used to characterize the effects of CMC incorporation and citric acid treatment on the chemical structure, surface composition, microstructure, and crystalline structure of BC. In addition, the overall performance of the composite films was evaluated in terms of rheological properties, mechanical strength, surface wettability, and water-management behavior, including water retention, re-hydration, and water vapor transmission performance.

## 2. Materials and Methods

### 2.1. Materials and Reagents

Sodium hydroxide, carboxymethyl cellulose(chemical pure, viscosity: 300–800 mPa·s, degree of substitution: 0.65–0.85, nominal molecular weight: 263.2), sodium hypophosphite, phosphate-buffered saline (PBS), absolute ethanol, sodium chloride, agar, glucose, yeast extract, peptone, disodium hydrogen phosphate dodecahydrate, and citric acid were purchased from Sinopharm Chemical Reagent Co., Ltd. (Shanghai, China). Universal bacterial primers (27F and 1492R) were purchased from Sangon Biotech Co., Ltd. (Shanghai, China). The Hestrin–Schramm (HS) liquid medium consisted of glucose (20.0 g/L), yeast extract (5.0 g/L), peptone (5.0 g/L), disodium hydrogen phosphate dodecahydrate (2.7 g/L), and citric acid (1.15 g/L). HS agar medium was prepared by supplementing the HS medium with 15 g/L agar. Kombucha was purchased from a local market (Xi’an, China).

### 2.2. Instruments

A Nicolet iS50 FTIR spectrometer (Thermo Fisher Scientific, Waltham, MA, USA), SU8600 field-emission scanning electron microscope (Hitachi, Tokyo, Japan), HH-4 thermostatic water bath (Shanghai Lichen Bangxi Instrument Technology Co., Ltd., Shanghai, China), D8 ADVANCE X-ray diffractometer (Bruker, Karlsruhe, Germany), DHR-1 rheometer (TA Instruments, New Castle, DE, USA), SDC-100H water contact angle meter (Puxi Precision Instrument Co., Ltd., Shanghai, China), DDL100 universal testing machine (DOLI, Munich, Germany), freeze dryer (Ningbo Scientz Freeze-Drying Equipment Co., Ltd., Ningbo, China), ZQZY-78CV shaking incubator (Zhichu Instrument Co., Ltd., Shanghai, China), ESCALAB QXi X-ray photoelectron spectrometer (Thermo Scientific, Waltham, MA, USA) and T100 PCR thermal cycler (Bio-Rad, Hercules, CA, USA) were used in this work.

### 2.3. Experimental Procedures

#### 2.3.1. Screening and Identification of BC-Producing Strains

Commercial kombucha was first activated by fermentation, and after a new thin pellicle formed on the liquid surface, 10% (*v*/*v*) kombucha broth was inoculated into sterilized HS medium and statically cultured for 5 days.

For strain isolation, 1 mL of the activated kombucha broth was added to 9 mL of 0.9% saline for pre-dilution, followed by serial tenfold dilution. Dilutions of 10^−3^, 10^−4^, and 10^−5^ were spread on HS agar plates and incubated statically at 30 °C for 48 h. Colony morphology, including size, transparency, and edge characteristics, was observed. Selected colonies were streaked on HS agar plates for purification, and single colonies were subsequently inoculated into HS liquid medium and sub-cultured twice in a ZQZY-78CV shaking incubator (Zhichu Instrument Co., Ltd., Shanghai, China) at 30 °C and 180 rpm.

Strain identification was performed according to the method of Wang et al. [28]. The DNA of the highest-BC-producing isolate was used as the template for PCR amplification with the universal primers 27F and 1492R. PCR amplification was performed using a T100 PCR thermal cycler (Bio-Rad, Hercules, CA, USA). The PCR products were sequenced by a commercial sequencing service provider. The obtained sequence was compared with the NCBI database, and a phylogenetic tree was constructed. The strain was identified as Novacetimonas hansenii and designated XNH6. Its BC dry weight reached 8.31 g/L and increased to 13.3 g/L after single-factor experiments and response surface optimization.

#### 2.3.2. Cultivation of XNH6 and Purification of BC

The XNH6 strain was removed from a −80 °C freezer and transferred into 30 mL of HS liquid medium. The culture was activated twice in a ZQZY-78CV shaking incubator (Zhichu Instrument Co., Ltd., Shanghai, China) at 30 °C and 180 rpm for 18 h each time to restore physiological activity. The activated broth was then inoculated into HS medium at 10% (*v*/*v*) and incubated at 30 °C for 6 days.

The XNH6-derived BC pellicles were collected from the HS liquid medium and washed in 0.1 mol/L sodium hydroxide solution using an HH-4 thermostatic water bath (Shanghai Lichen Bangxi Instrument Technology Co., Ltd., Shanghai, China) at 80 °C to remove residual fermentation broth. The washing solution was replaced according to the color change until the surface residues were completely removed. The films were finally rinsed thoroughly with distilled water until the pH became neutral, yielding purified BC films.

#### 2.3.3. Preparation of BC/CMC Composite Films

CMC was added to HS liquid medium at concentrations of 0.2, 0.4, 0.6, 0.8, and 1.0 g/L, respectively, and sterilized together with the medium. The activated culture was inoculated at 10% (*v*/*v*) into Petri dishes containing 20 mL of liquid medium, statically cultured at 30 °C for 6 days, and then washed repeatedly with distilled water. The films were designated BC/CMC-1, BC/CMC-2, BC/CMC-3, BC/CMC-4, and BC/CMC-5 (Table 1). The CMC concentration range was selected based on preliminary experiments. CMC concentrations below 0.2 g/L showed limited effects on BC network regulation, whereas concentrations above 1.0 g/L increased medium viscosity and restricted oxygen transfer and nutrient diffusion, thereby inhibiting BC pellicle formation. Therefore, 0.2–1.0 g/L CMC was selected for subsequent experiments.

#### 2.3.4. Citric Acid Post-Treatment of BC/CMC Composite Films

To improve structural stability, a 10% citric acid solution was prepared and supplemented with sodium hypophosphite at 1% of the citric acid mass. Sodium hypophosphite was used as a catalyst to facilitate possible esterification between citric acid and hydroxyl-containing polysaccharide chains during heat treatment. The BC/CMC composite films prepared in Section 2.3.3 were immersed in the citric acid solution containing sodium hypophosphite, gently stirred for 1 h, and then heated at 140 °C for 15 min. During heating, the multiple carboxyl groups of citric acid may react with the hydroxyl groups of BC and CMC, potentially contributing to ester-linkage-assisted stabilization of the composite network. After heat treatment, the films were immersed in 0.1 mol/L sodium hydroxide solution in an HH-4 thermostatic water bath (Shanghai Lichen Bangxi Instrument Technology Co., Ltd., Shanghai, China) at 80 °C for 2 h, and the washing solution was replaced every 30 min. The films were subsequently soaked and rinsed thoroughly with distilled water until the pH reached neutrality, providing purified BC/CMC composite films with different CMC concentrations.

### 2.4. Characterization of BC/CMC Composite Films

#### 2.4.1. FTIR

Freeze-dried BC and BC/CMC films were analyzed using a Nicolet iS50 FTIR spectrometer (Thermo Fisher Scientific, Waltham, MA, USA) equipped with an ATR detector in attenuated total reflectance mode. The spectra were collected over the range of 4000–600 cm^−1^ with a resolution of 4 cm^−1^ and 32 scans [29].

#### 2.4.2. XPS

XPS was performed to analyze the surface elemental composition and chemical bonding states of BC, BC/CMC-3, and BC/CMC-4 films using an ESCALAB QXi X-ray photoelectron spectrometer (Thermo Scientific, Waltham, MA, USA) equipped with a monochromatic Al Kα radiation source (1486.6 eV). Charging effects were compensated using a flood gun. The pass energy was 200 eV for survey spectra and 20 eV for high-resolution spectra [30]. The binding energy scale was calibrated by setting the C–C/C–H component of the C 1s peak to 284.8 eV. The high-resolution C 1s spectra were fitted using Thermo Scientific Avantage version v5.99 software, and the relative contents of C 1s components were calculated from the fitted peak areas.

#### 2.4.3. XRD

The crystalline structures of BC and BC/CMC composite films were analyzed using a D8 ADVANCE X-ray diffractometer (Bruker, Karlsruhe, Germany). The scanning range was 5–50°, the scanning rate was 5°/min, the step size was 0.02°, the current was 30 mA, the voltage was 40 kV, and Cu-Kα radiation (λ = 0.15418 nm) was used. Prior to crystallinity calculation, background subtraction was performed for all XRD patterns using the same processing parameters. The CrI was calculated by the peak-area integration method using Origin 2024 software. The crystalline peak area was obtained from the fitted cellulose I diffraction peaks near 14°, 16°, and 22°, and the total diffraction area was obtained from the fitted XRD profile within the scanning range of 5–50°. The CrI was calculated according to Equation (1). [31].

(1) $$CrI\left(\%\right)=\frac{\Sigma C}{\Sigma T}\times 100\%$$

where ΣC is the total area of the main crystalline peaks of cellulose I near 14°, 16°, and 22°, and ΣT is the total diffraction area obtained from the fitted XRD profile within the same scanning range.

#### 2.4.4. SEM

Freeze-dried BC and BC/CMC films were cut into small pieces (approximately 0.5 cm × 0.5 cm) and fixed onto the sample stage. The sample surfaces were sputter-coated with gold to improve conductivity prior to imaging. The samples were then observed using an SU8600 field-emission scanning electron microscope (Hitachi, Tokyo, Japan) under an accelerating voltage of 5 kV [32].

#### 2.4.5. Water Retention and Re-Hydration Measurements

For the water-retention test, the initial mass of the wet composite film was recorded as M0. The samples were placed in a constant temperature and humidity chamber maintained at 25 °C and 70% relative humidity. The films were removed every 4 h and weighed; the mass at time t was recorded as Mt. The water retention ratio was calculated according to Equation (2) [33].

(2) $$Water\;retention\;ratio\left(\%\right)=\frac{M_{t}}{M_{0}}\times 100\%$$

where M_t_ is the film mass at time t (g) and M_0_ is the initial mass of the wet film (g).

For the re-hydration test, BC composite films were freeze-dried using a freeze dryer (Ningbo Scientz Freeze-Drying Equipment Co., Ltd., Ningbo, China), cut into square specimens (5 cm × 5 cm), and the initial mass was recorded as W0. The samples were immersed in excess deionized water and re-hydrated at room temperature in sealed conditions. At predetermined time intervals (1, 2, 4, 8, 16, and 32 h), the samples were removed, gently blotted with filter paper to remove surface water, and weighed as W_t_. The swelling ratio was calculated according to Equation (3).

(3) $$Swelling\;ratio\left(\%\right)=\frac{W_{t}-W_{0}}{W_{0}}\times 100\%$$

where W_t_ is the film mass at time t (g) and W_0_ is the initial mass of the freeze-dried film (g).

#### 2.4.6. Water Vapor Transmission Rate (WVTR)

The WVTR was determined by a gravimetric method modified from the cup method [34]. Briefly, 10 mL of distilled water was added to a centrifuge tube with a diameter of 1.5 cm. Freeze-dried BC and BC/CMC films were cut into circular specimens with a thickness of 0.2 mm and a size slightly larger than the tube opening, placed over the opening, and sealed tightly with sealing film. The initial weight of the assembly was recorded as M0. The device was then placed in a constant temperature and humidity chamber at 37 °C and 35% relative humidity for 24 h. After the test, the final mass was recorded as M, and WVTR was calculated according to Equation (4).

(4) $$WVTR=\frac{M_{0}-M}{A\times 24}$$

where M_0_ is the initial mass of the centrifuge-tube assembly (g), M is the final mass after 24 h (g), and A is the area of the tube opening (m^2^).

#### 2.4.7. Water Contact Angle

Water contact angle was evaluated using an SDC-100H water contact angle meter (Puxi Precision Instrument Co., Ltd., Shanghai, China), and the droplet-spreading method was applied to freeze-dried BC and BC/CMC films. Freeze-dried BC and BC/CMC films were placed on glass slides, and a 5 μL droplet of deionized water was deposited onto the film surface using a micro-syringe. Three different surface regions were measured for each sample, and the contact angle formed at the solid–liquid interface was recorded [35].

#### 2.4.8. Rheological Measurements

Rheological behavior was evaluated using a DHR-1 rheometer (TA Instruments, New Castle, DE, USA). Wet film specimens with a diameter of 20 mm and a thickness of 1 mm were tested using a 20 mm parallel-plate geometry. Before measurement, the samples were equilibrated at 25 °C for 5 min. The shear rate was increased from 0.01 s^−1^ to 100 s^−1^, and the corresponding shear stress and apparent viscosity were recorded [36].

#### 2.4.9. Mechanical Properties

To evaluate tensile properties, wet BC and BC/CMC composite films with different CMC concentrations were cut into strips with dimensions of 40 mm × 10 mm × 1 mm. Each specimen was mounted carefully between the upper and lower grips of a DDL100 universal testing machine (DOLI, Munich, Germany) without pre-stress. The initial gauge length was 10 mm. The samples were stretched at a constant rate of 10 mm/min until fracture, and the stress–strain data were recorded automatically during testing [37].

### 2.5. Statistical Analysis

The water-management tests were performed with n = 3 independent samples, and the results were expressed as mean ± standard deviation. For water contact angle measurements, three films were tested for each group, and three different points were measured on each film. For fiber diameter analysis, at least 200 fibers were measured for each group. Statistical analysis was conducted using SPSS 27.0 software by one-way analysis of variance (ANOVA), and significant differences were determined by Duncan’s multiple range test at *p* < 0.05. Figures were prepared using Origin 2024 software. The phylogenetic tree was constructed using MEGA 12 software. BC fiber diameters were measured using Nano Measurer, and water contact angles were analyzed using the Contact Angle plug-in in ImageJ version 1.53.

## 3. Results

### 3.1. Isolation and Identification of the BC-Producing Strain

**Figure 1:**
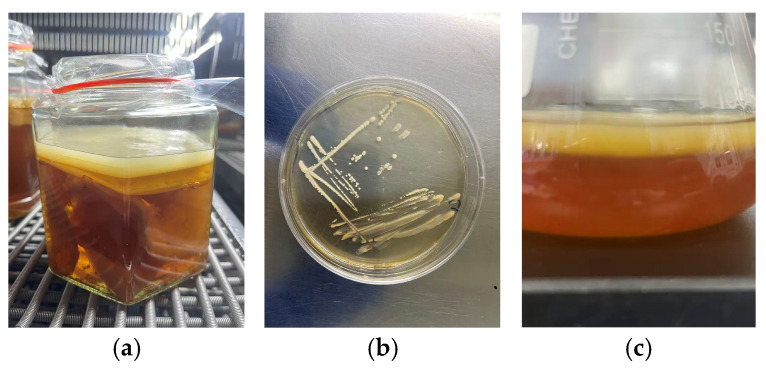
Isolation and identification of BC-producing strains: (**a**) Kombucha, (**b**) colony and (**c**) BC.

**Figure 2:**
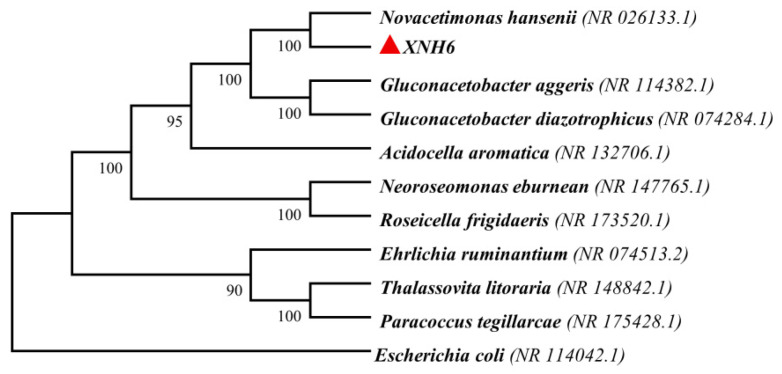
Phylogenetic tree of the selected BC-producing strain XNH6.

After dilution, the kombucha broth (Figure 1a) was spread on HS agar plates. Following spread plating and streak purification, colonies with gel-like margins were observed (Figure 1b). A single colony was inoculated into HS liquid medium, and after 3 days of cultivation, a bacterial cellulose pellicle formed at the air–liquid interface (Figure 1c). The strain sequence was then determined and used to construct a phylogenetic tree. According to the phylogenetic relationship shown in Figure 2, the strain was identified as *Novacetimonas hansenii* and designated XNH6. Its dry BC yield reached 8.31 g/L and increased to 13.3 g/L after single-factor experiments and response surface optimization.

### 3.2. Structural and Functional Characterization of BC/CMC Composite Films

**Figure 3:**
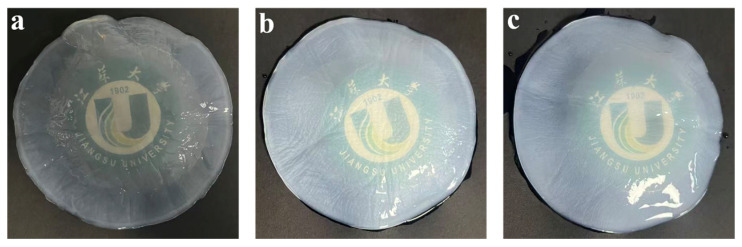
Representative macroscopic appearances of wet BC and selected BC/CMC composite films: (**a**) BC, (**b**) BC/CMC-3, and (**c**) BC/CMC-4.

Representative photographs of neat BC and selected BC/CMC composite films are shown in Figure 3. All three wet films showed continuous film morphology, and the BC/CMC composite films maintained an intact appearance comparable to that of neat BC.

#### 3.2.1. FTIR Analysis

**Figure 4:**
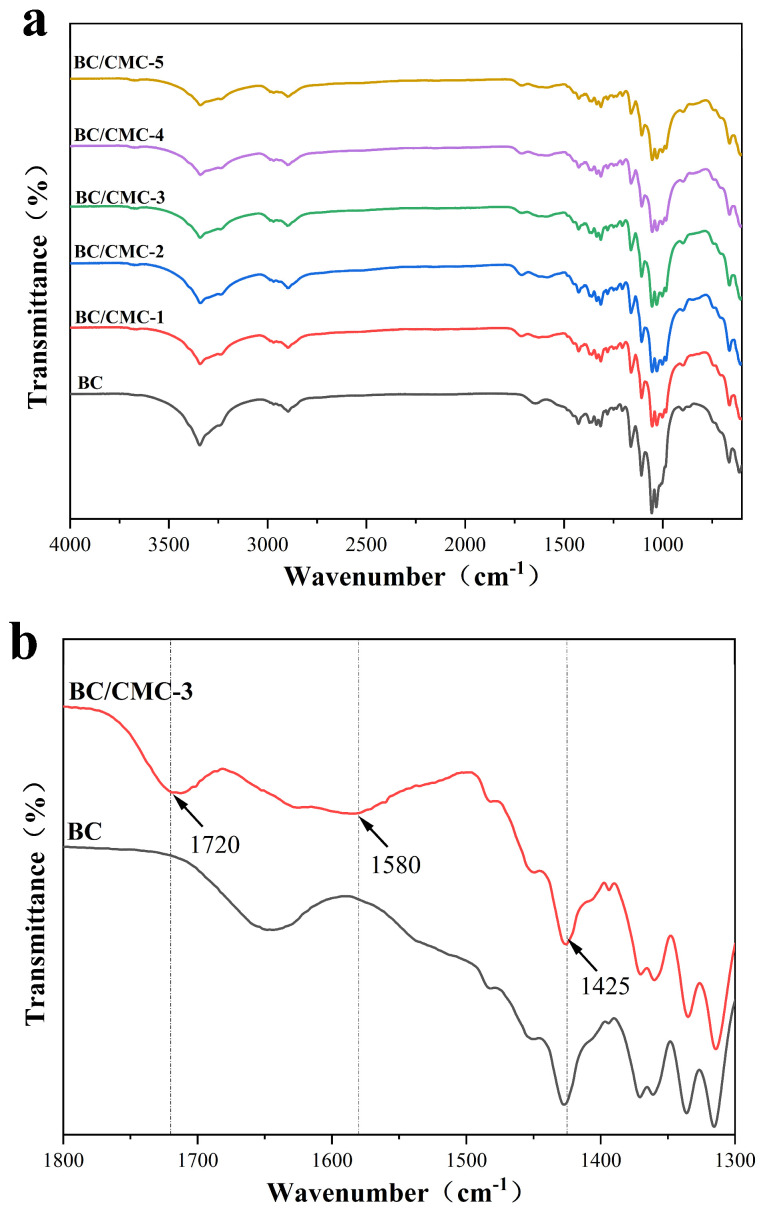
FTIR spectra of BC/CMC composite films: (**a**) full spectra; (**b**) enlarged local region.

As shown in Figure 4a, neat BC exhibited a broad and intense absorption band at approximately 3400 cm^−1^ corresponding to O–H stretching, a characteristic peak at around 2900 cm^−1^ assigned to C–H stretching, and absorption bands in the range of 1160–1030 cm^−1^ attributed to C–O–C and C–O vibrations in the cellulose backbone. Compared with neat BC, the BC/CMC composite films retained these characteristic absorption bands, indicating that neither CMC incorporation nor citric acid treatment destroyed the fundamental cellulose skeleton of BC. Notably, as shown in Figure 4b, a new absorption band appeared at approximately 1720 cm^−1^ in the composite films, which can be assigned to the C=O stretching vibration of ester groups, suggesting possible esterification during citric acid treatment [37]. However, this band alone cannot be used as conclusive evidence for the degree of cross-linking. Therefore, in this study, the 1720 cm^−1^ band was interpreted as qualitative evidence of possible ester-linkage formation and was discussed together with XPS results and macroscopic property changes. In addition, the enhanced absorption bands near 1580 cm^−1^ and 1425 cm^−1^, corresponding to the asymmetric and symmetric stretching vibrations of COO− groups in CMC, indicated that CMC was successfully introduced into the BC network. The broadening and slight shift in the O–H stretching band further suggest enhanced hydrogen-bond interactions between BC and CMC [22].

#### 3.2.2. XPS Analysis

**Figure 5:**
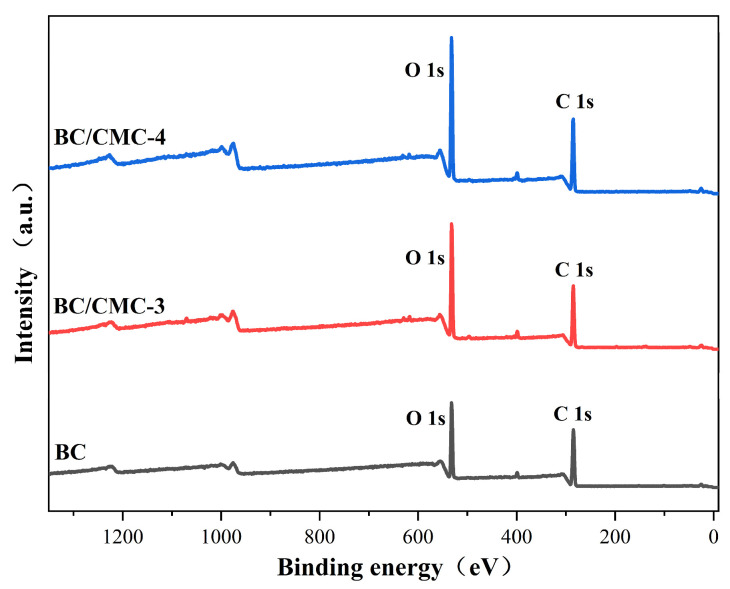
XPS survey spectra of BC, BC/CMC-3, and BC/CMC-4 films.

To analyze the effect of CMC incorporation on the surface elemental composition and functional group structure of BC films, XPS analysis was performed on BC, BC/CMC-3, and BC/CMC-4 films, as shown in Figure 5 and Table 2. The XPS survey spectra showed that C 1s and O 1s signals were mainly detected on the surfaces of all three samples, indicating that C and O were the predominant surface elements. As shown in Table 2, the atomic percentages of C and O in the BC film were 75.45% and 22.01%, respectively, with an O/C ratio of 0.292. Compared with BC, the O atomic percentages of BC/CMC-3 and BC/CMC-4 increased to 32.63% and 33.47%, respectively, while their O/C ratios increased to 0.513 and 0.524, respectively, indicating an increased oxygen content on the surface of the composite films after CMC incorporation. The high-resolution C 1s spectra were further deconvoluted. The relative contents of C–C/C–H, C–O/C–O–C, O–C–O/C=O, and O–C=O components in BC were 38.71%, 47.15%, 12.12%, and 2.00%, respectively. The corresponding values were 23.30%, 57.18%, 16.73%, and 2.79% for BC/CMC-3, and 23.38%, 55.39%, 19.73%, and 1.49% for BC/CMC-4. Compared with BC, both BC/CMC-3 and BC/CMC-4 showed a decrease in the C–C/C–H component and increases in the C–O/C–O–C and O–C–O/C=O components [38].

Overall, the XPS results indicate that CMC incorporation altered the surface elemental composition and C 1s chemical bonding structure of BC films, mainly reflected by the increased O/C ratio and the increased proportion of oxygen-containing carbon components.

#### 3.2.3. XRD Analysis

**Figure 6:**
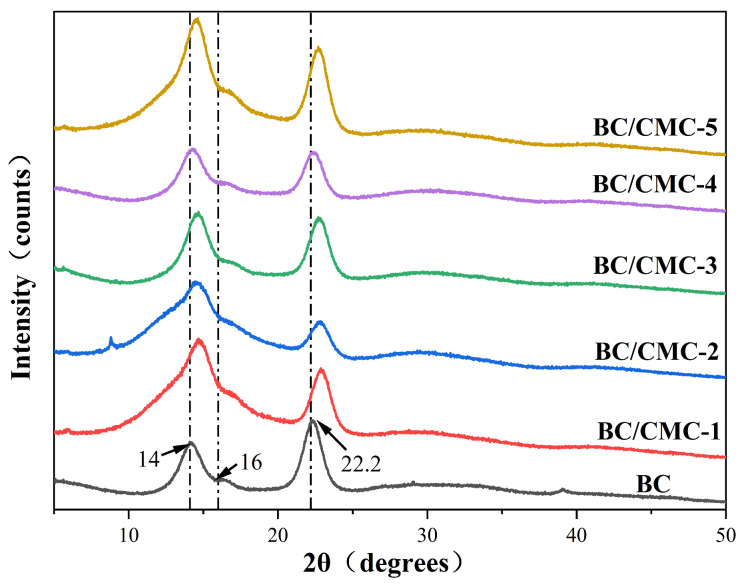
XRD patterns of BC/CMC composite films.

The XRD patterns of BC and BC/CMC composite films are shown in Figure 6. Neat BC displayed three characteristic diffraction peaks at approximately 14.0°, 16.0°, and 22.2°, corresponding to the (101), (110), and (200) crystallographic planes of cellulose I, respectively [38]. Compared with neat BC, all BC/CMC composite films retained these characteristic peaks, indicating that the introduction of CMC and subsequent citric acid treatment did not change the main crystal form of BC. However, systematic shifts in peak positions were observed. For example, the diffraction angle of the (200) plane increased from 22.2° for BC to 22.43–22.85° for the composite films. According to Bragg’s law (2d sin θ = nλ), an increase in diffraction angle indicates a decrease in interplanar spacing (d), suggesting that CMC incorporation and citric acid treatment altered the interactions among BC chains and thereby affected the arrangement of the micro-crystalline regions [22].

The crystallinity of each sample was further calculated, and the results are summarized in Table 3. Neat BC exhibited a high crystallinity of 87.49%, which is consistent with the characteristic features of bacterial cellulose reported in the literature [39]. After CMC incorporation, the crystallinity of all BC/CMC composite films decreased to the range of 77.69–82.57%, indicating that CMC disturbed the regular packing of BC molecular chains and reduced crystalline order to some extent. BC/CMC-3 exhibited the lowest crystallinity, suggesting that this CMC dosage caused the strongest disruption to the arrangement of BC crystalline domains. Notably, the crystallinity did not vary monotonically with increasing CMC concentration, implying that the effect of CMC on crystalline order may be jointly regulated by concentration, intermolecular interactions, and possible ester-linkage-assisted network stabilization. It should be noted that the CrI values obtained from XRD are method-dependent and should be regarded as apparent crystallinity indices for relative comparison among samples analyzed under identical conditions, rather than absolute crystallinity values.

#### 3.2.4. SEM Analysis

**Figure 7:**
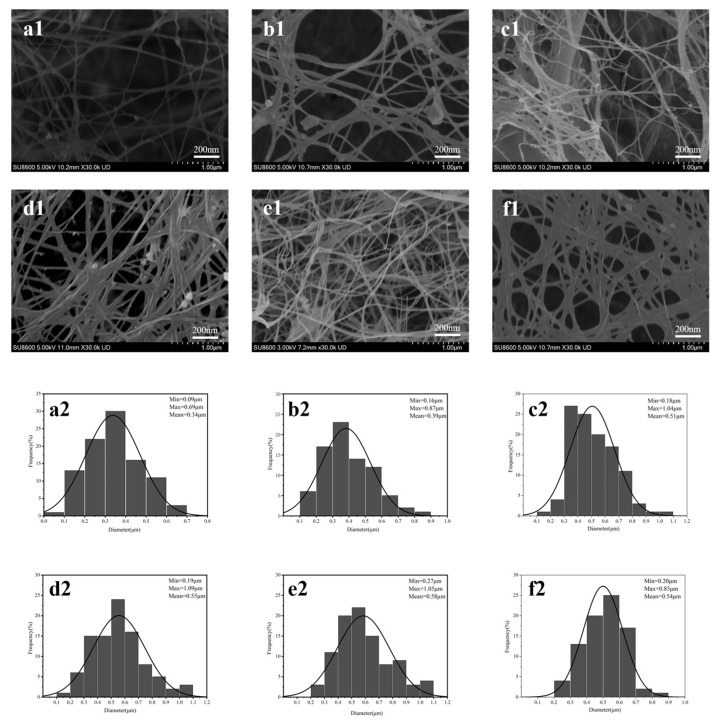
SEM images (**a1**–**f1**) and fiber diameter distributions (**a2**–**f2**) of BC and BC/CMC composite films.

Figure 7 presents the SEM micrographs and corresponding fiber diameter histograms of BC and BC/CMC composite films prepared with different CMC concentrations. Neat BC exhibited a typical three-dimensional nanofibrous network, with relatively loose fiber entanglement and clearly visible nanoscale pores (Figure 7a1). The fiber diameter distribution (Figure 7a2) showed that the BC fibers were mainly distributed in the range of 0.09–0.69 μm, with an average diameter of 0.34 μm, which is characteristic of bacterial cellulose nanofibers.

Compared with neat BC, the overall microstructure of the BC/CMC composite films gradually evolved from a loose network to a denser network with increasing CMC concentration. Starting from BC/CMC-1, the degree of fiber interweaving increased and the inter-fiber pores gradually decreased; this change became particularly pronounced in BC/CMC-3 and BC/CMC-4. The fiber diameter distributions further confirmed this trend. The average apparent fiber diameters of BC/CMC-1, BC/CMC-2, BC/CMC-3, BC/CMC-4, and BC/CMC-5 increased to 0.39, 0.51, 0.55, 0.58, and 0.54 μm, respectively, indicating that the introduction of CMC promoted an increase in the apparent diameter of BC fibers.

#### 3.2.5. Water Retention and Re-Hydration Properties

**Figure 8:**
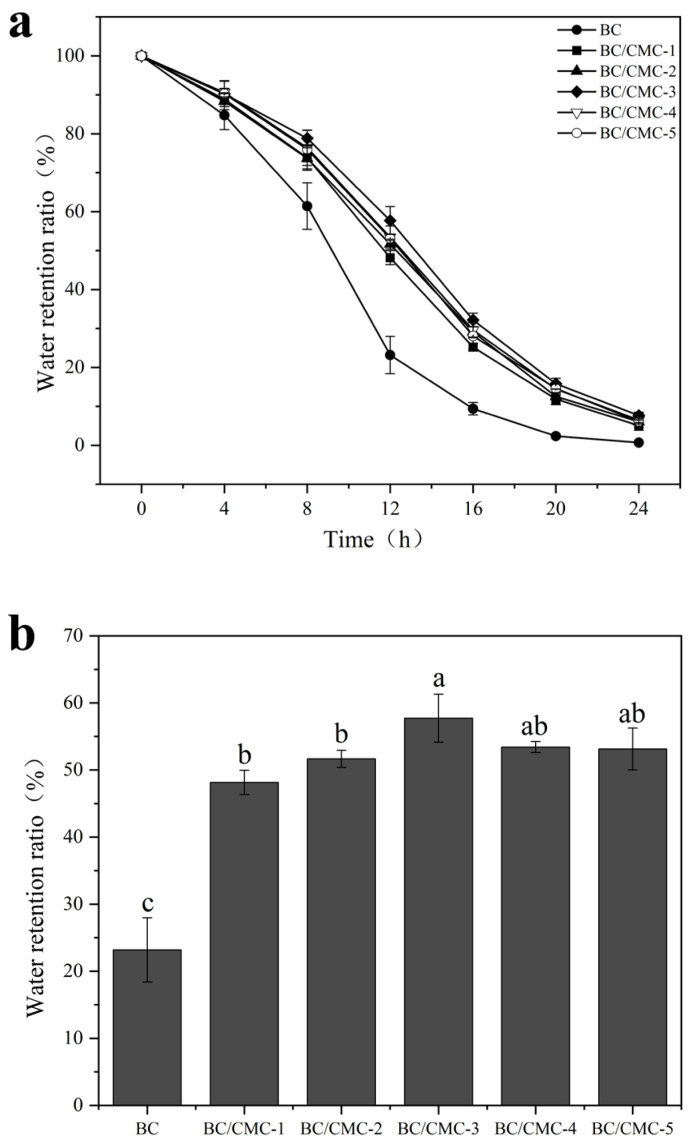
Water-retention ratios of BC/CMC composite films: (**a**) full water-retention curves; (**b**) water-retention ratio at 12 h. Data that are significant different (*p* < 0.05) are denoted with different letters in the figure.

To evaluate the effect of CMC concentration on the water-retention behavior of wet BC/CMC composite films, the water retention ratio of each sample was determined. As shown in Figure 8a, the water retention ratio of both BC and BC/CMC films gradually decreased with time. However, compared with neat BC, all composite films maintained generally higher water retention throughout the test period, indicating that the introduction of CMC effectively improved the water-holding capacity of the films. Among them, BC/CMC-3 exhibited superior water-retention performance at most time points, especially during the intermediate dehydration stage. At 12 h (Figure 8b), the water retention ratio of neat BC was 23.18 ± 4.79%, whereas that of BC/CMC-3 reached 57.72 ± 3.57%, which was approximately 2.49 times that of BC.

**Figure 9:**
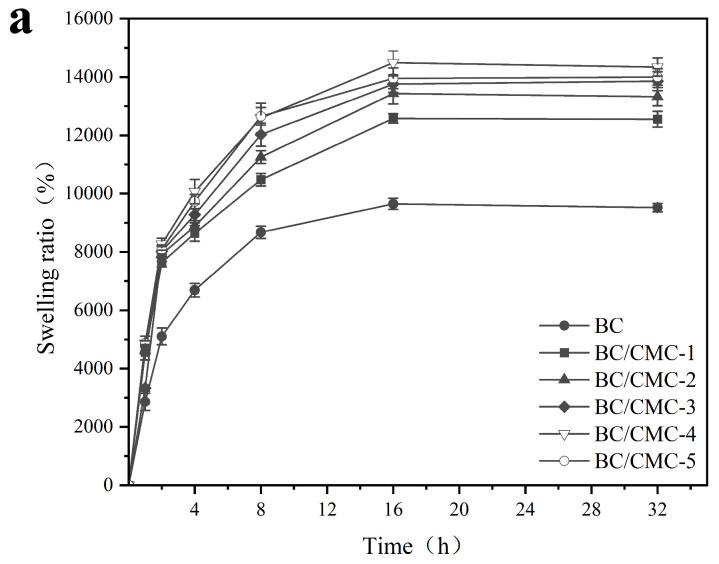

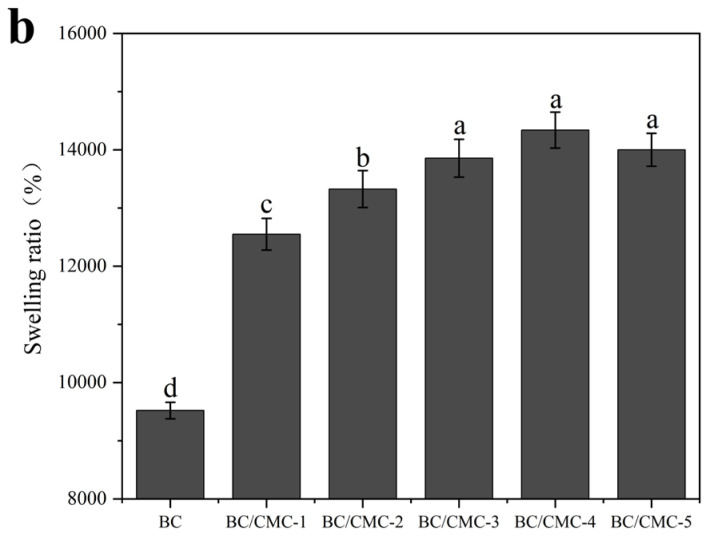
Re-hydration ratios of BC/CMC composite films: (**a**) full re-hydration curves; (**b**) re-hydration ratio at 32 h. Data that are significant different (*p* < 0.05) are denoted with different letters in the figure.

To further evaluate the effect of different CMC concentrations on the re-hydration of freeze-dried BC/CMC composite films, the re-hydration ratio of each sample was measured. As shown in Figure 9a, the re-hydration ratio of both BC and BC/CMC films increased with time and displayed a typical two-stage re-hydration profile. In the first stage (0–8 h), the re-hydration ratio increased rapidly, indicating that water could quickly penetrate the film surface and shallow pores. In the second stage (8–32 h), the increase gradually slowed, and the values became stable from 16 to 32 h (*p* > 0.05), indicating that the samples approached water-absorption equilibrium as water diffused further into the inner structure. BC/CMC-4 showed the best re-hydration behavior at most time points. At 1, 2, and 4 h, the re-hydration ratios of BC/CMC-4 were approximately 4860 ± 249%, 8268 ± 203%, and 10,069 ± 419%, respectively, all significantly higher than those of BC (2860 ± 297%, 5105 ± 289%, and 6691 ± 233%, respectively; *p* < 0.05). At 32 h (Figure 9b), the re-hydration ratio of BC was approximately 9519 ± 142%, whereas BC/CMC-4 reached 14,001 ± 283%, representing a significant increase of approximately 47.09% (*p* < 0.05).

#### 3.2.6. Water Vapor Transmission Rate

**Figure 10:**
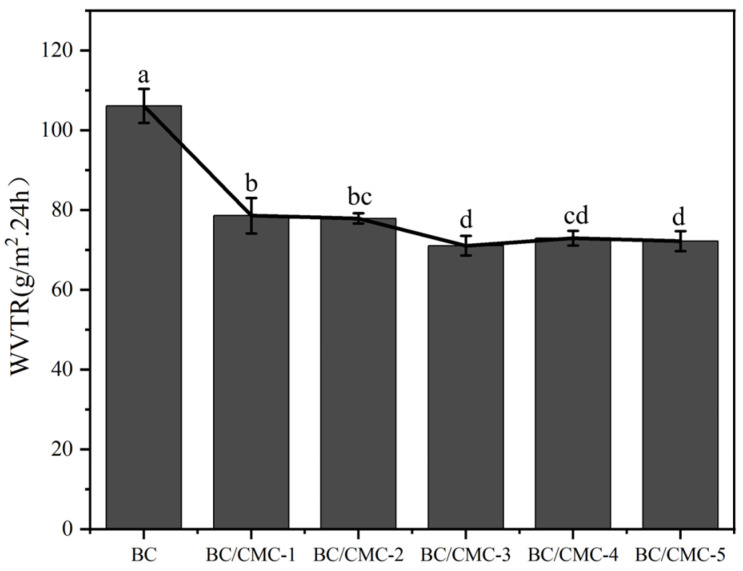
Water vapor transmission rates of BC/CMC composite films. Data that are significant different (*p* < 0.05) are denoted with different letters in the figure.

As shown in Figure 10, neat BC exhibited the highest WVTR, approximately 106.13 ± 4.24 g/(m^2^·24 h). After CMC incorporation and citric acid post-treatment, the WVTR values of all BC/CMC composite films decreased to the range of 71–79 g/(m^2^·24 h), corresponding to reductions of approximately 25–33% relative to neat BC. This result indicates that composite formation improved the water vapor barrier performance of the films. Among all samples, BC/CMC-3 exhibited the lowest WVTR, approximately 71.07 ± 2.46 g/(m^2^·24 h), which was about 33% lower than that of neat BC.

#### 3.2.7. Water Contact Angle Analysis

**Figure 11:**
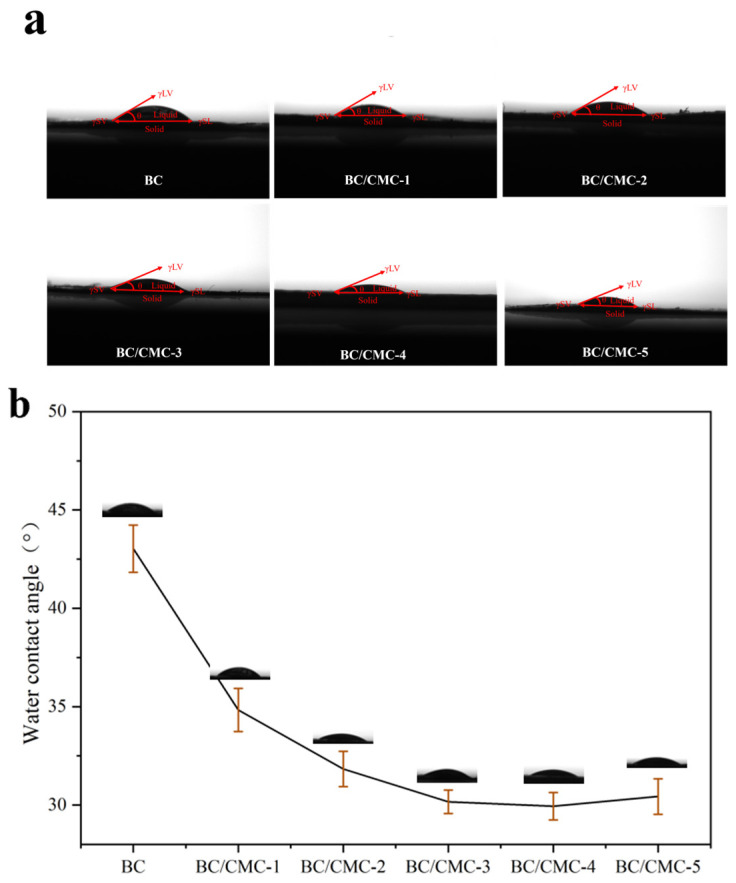
Water contact angle images (**a**) and contact angle values (**b**) of BC/CMC composite films.

To evaluate the effect of CMC concentration on the surface wettability of BC/CMC composite films, water contact angles were measured. As shown in Figure 11, neat BC exhibited a water contact angle of approximately 43.0 ± 1.2°, indicating moderate hydrophilicity. After CMC incorporation, the water contact angles of the BC/CMC composite films decreased overall to the range of 29–35°. Specifically, the values for BC/CMC-1, BC/CMC-2, BC/CMC-3, BC/CMC-4, and BC/CMC-5 were approximately 34.8 ± 1.1°, 31.8 ± 0.9°, 30.2 ± 0.6°, 30.0 ± 0.7°, and 30.4 ± 0.9°. These results show that all samples exhibited hydrophilic surfaces, and the incorporation of CMC resulted in a moderate decrease in water contact angle compared with neat BC.

#### 3.2.8. Rheological Properties

**Figure 12:**
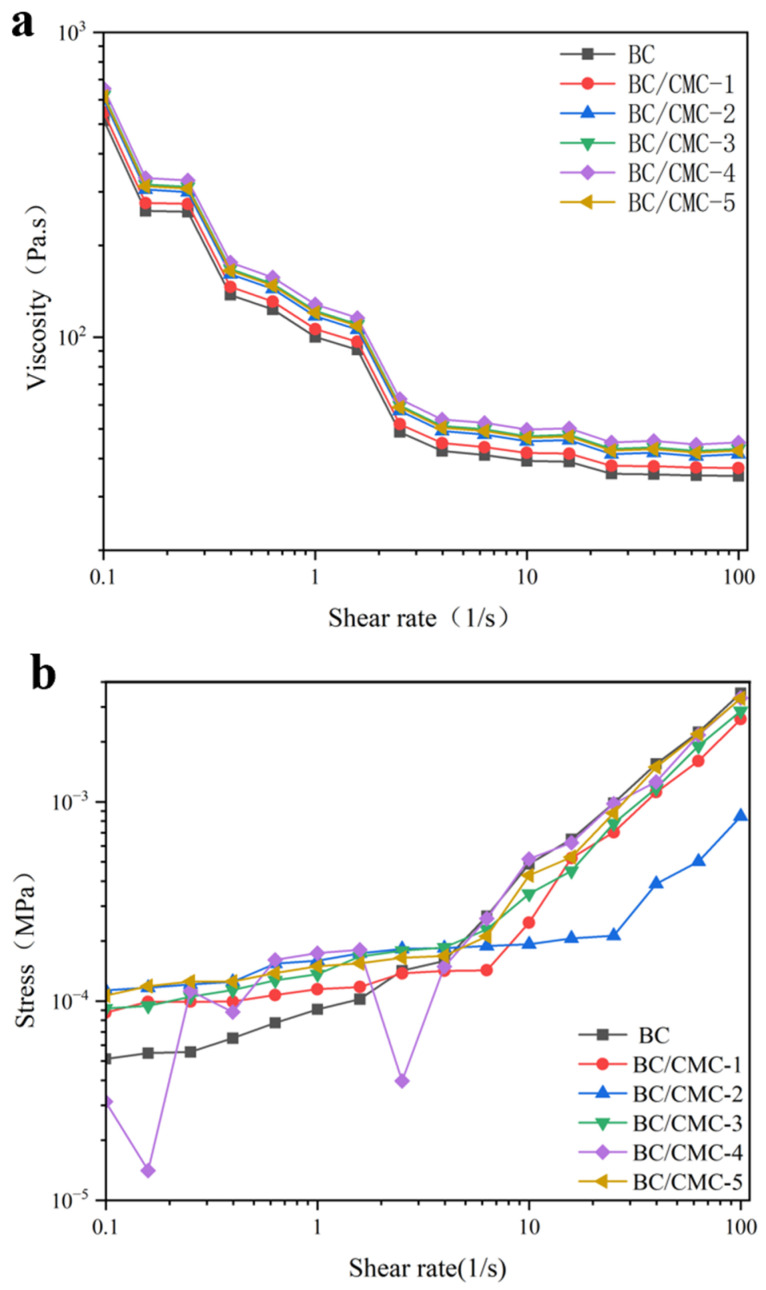
Rheological behavior of BC/CMC composite films: (**a**) shear rate-viscosity curves; (**b**) shear rate-shear stress curves.

To investigate the effect of CMC incorporation on the steady-shear behavior of BC/CMC composite films, shear rheological measurements were performed. As shown in Figure 12a, the apparent viscosity of neat BC and all BC/CMC composite films decreased with increasing shear rate, indicating pronounced shear-thinning behavior. For neat BC, the apparent viscosity dropped rapidly from a relatively high level at low shear rates to a much lower value at 100 s^−1^, suggesting progressive orientation and rearrangement of the internal structure under external shear. Compared with neat BC, all BC/CMC composite films exhibited higher apparent viscosity over the entire test range, with BC/CMC-4 showing the most pronounced effect. At 0.1, 1.0, and 100 s^−1^, the apparent viscosities of BC/CMC-4 were 653.74, 128.01, and 45.13 Pa·s, respectively, all higher than those of neat BC, indicating that CMC incorporation increased flow resistance under steady shear [40].

The shear stress results further supported this trend (Figure 12b). With increasing shear rate, the shear stress of all samples generally increased, and the BC/CMC composite films consistently showed higher values than neat BC. At 1 s^−1^, the shear stress of neat BC was approximately 90.9 Pa, whereas that of BC/CMC-4 increased to approximately 174 Pa, about 1.91 times that of BC. This result suggests that an appropriate amount of CMC enhances the ability of the composite films to resist deformation under external force.

#### 3.2.9. Mechanical Properties Analysis

**Figure 13:**
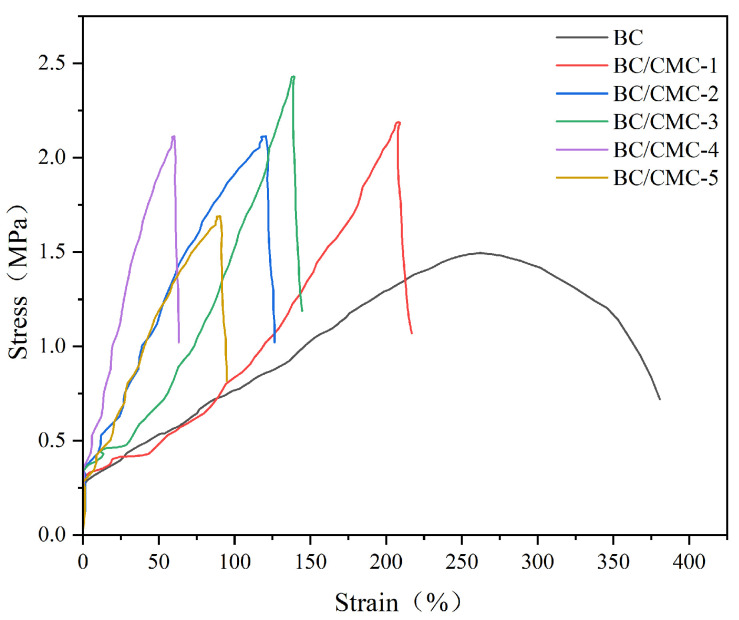
Tensile properties of BC/CMC composite films.

As shown in Figure 13, neat BC exhibited relatively low fracture strength but high elongation at break. After in situ compositing with CMC and subsequent citric acid post-treatment, the stress–strain curves of all composite films shifted toward higher stress regions, indicating improved tensile strength [26]. Among them, BC/CMC-3 exhibited the highest fracture strength, approximately 2.4 MPa, which was about 1.7 times higher than that of neat BC, indicating that an appropriate amount of CMC improved the load-bearing capacity of the films.

In contrast to the trend in strength, the elongation at break of the composite films was generally lower than that of neat BC. The elongation at break of BC was 353.05%, whereas that of the BC/CMC composite films ranged from 66.3% to 172.1%, indicating that the extensibility of the materials decreased while tensile strength increased. In addition, the slope of the initial linear region of the stress–strain curves suggests that the elastic modulus of the composite films was generally higher than that of neat BC, indicating increased stiffness. These results demonstrated that CMC incorporation and citric acid post-treatment contributed to the formation of a stronger but more rigid network, possibly through hydrogen bonding, ester-linkage-assisted stabilization, and network densification.

## 4. Discussion

This study demonstrated that introducing CMC during BC biosynthesis, followed by citric acid post-treatment, can significantly regulate the microstructure and macroscopic properties of the composite films without destroying the native cellulose I crystal form of BC [22,26]. FTIR analysis showed that, besides retaining the characteristic absorption bands of the cellulose backbone, the BC/CMC composite films exhibited a new band near 1720 cm^−1^ and enhanced bands near 1580 and 1425 cm^−1^, indicating successful CMC incorporation and possible ester-linkage formation after citric acid treatment [41,42]. XPS analysis further supported the incorporation of CMC into the BC network, as the BC/CMC films showed higher O/C ratios and increased C–O/C–O–C and O–C–O/C=O components compared with neat BC. These changes indicate an increase in oxygen-containing carbon structures associated with CMC, providing additional evidence for the construction of BC/CMC composite networks [43]. It should be emphasized that the FTIR band near 1720 cm^−1^ and the XPS changes provide qualitative evidence for possible ester-linkage-assisted stabilization, but they do not quantify the degree of cross-linking. Thus, the improved film properties are discussed here as the combined result of CMC incorporation, hydrogen-bond interactions, possible ester-linkage-assisted stabilization, and network densification. XRD analysis showed that all composite films retained the characteristic diffraction peaks of cellulose I, but their crystallinity decreased compared with that of BC, indicating that CMC disturbed the regular packing of BC molecular chains to some extent [12]. SEM further showed that increasing CMC concentration increased the apparent fiber diameter, reduced pore size, and promoted gradual densification of the network, confirming that CMC participated in the construction and reorganization of the BC fiber network [44].

In terms of water-management performance, the introduction of CMC significantly improved both water retention and re-hydration, but the optimal samples for these two properties were not identical [22,45]. BC/CMC-3 showed the best water-retention behavior, whereas BC/CMC-4 exhibited the highest re-hydration capacity. This result indicates that although both properties are related to hydrophilicity and network structure, they are governed by different dominant factors. Water retention depends more strongly on the ability of the network to bind and retain water stably, whereas re-hydration is more strongly influenced by hydrophilic-group content, pore structure, and the capacity of the freeze-dried network to re-swell [23,46]. Meanwhile, the water contact angle results showed that neat BC was already highly hydrophilic, and CMC incorporation moderately reduced the contact angle from 43.0 ± 1.2° to 29–35°. Therefore, the contact-angle results should be regarded as supporting evidence of maintained and slightly enhanced surface hydrophilicity, rather than the main factor determining the overall water-management performance. The superior re-hydration of BC/CMC-4 may be more closely associated with its higher CMC content, enriched hydrophilic groups, and greater re-swelling capacity of the freeze-dried network [47,48].

The WVTR results showed that all BC/CMC composite films exhibited lower water vapor transmission rates than neat BC, indicating that CMC incorporation and subsequent citric acid post-treatment contributed to improved water vapor barrier performance, probably because of the denser internal network and increased vapor-diffusion tortuosity. [27]. Combined with the SEM results, this suggests that the denser internal network increased the resistance to water vapor diffusion and thus reduced WVTR. BC/CMC-3 exhibited the lowest WVTR, suggesting that this composition achieved a better balance between network densification and restriction of vapor-transport channels [49]. Significantly, although the composite films became more hydrophilic at the surface, WVTR did not increase, indicating that surface wettability and water vapor transport are not governed by exactly the same structural factors; the latter depends more strongly on the continuity of the internal network and pore characteristics [47].

The rheological and mechanical results further demonstrated that CMC incorporation strengthened intermolecular interactions within the composite films and improved network stability [26]. All composite films exhibited higher apparent viscosity and shear stress than neat BC, indicating stronger resistance to flow-induced deformation under shear. Tensile testing showed that the fracture strength and stiffness of the composite films increased overall, whereas the elongation at break decreased, suggesting that the original loose BC network was converted into a denser and more rigid load-bearing structure [44]. Among the tested samples, BC/CMC-3 showed the best mechanical strength, indicating that a moderate amount of CMC is favorable for balancing enhanced chain interactions with coordinated network deformation.

The difference between BC/CMC-3 and BC/CMC-4 suggests that mechanical/barrier properties and re-hydration/wettability are controlled by different dominant factors. BC/CMC-3 may represent an optimal balance between CMC incorporation and network stabilization, resulting in a denser and more continuous structure that favors stress transfer and restricts water vapor diffusion. In contrast, BC/CMC-4 contains a higher amount of CMC, providing more hydrophilic carboxymethyl and hydroxyl groups that facilitate water penetration and re-swelling of the freeze-dried network. However, the higher hydrophilicity and swelling tendency of BC/CMC-4 may weaken its effective compactness and barrier performance. Therefore, BC/CMC-3 showed better mechanical and WVTR performance, whereas BC/CMC-4 exhibited superior re-hydration and wettability.

Overall, different CMC concentrations imparted distinct performance advantages to the composite films. BC/CMC-3 performed better in water retention, water vapor barrier performance, and mechanical strength, making it more suitable for applications requiring structural stability and moisture regulation. By contrast, BC/CMC-4 exhibited superior re-hydration behavior and surface wettability, suggesting greater suitability for material systems that require rapid water uptake and re-swelling. Therefore, the present study demonstrated that the structure and performance of BC/CMC composite films can be effectively regulated by adjusting the CMC dosage, providing a useful basis for their development as moisture-management materials. From an application perspective, BC/CMC-3 may be more suitable for moisture-regulating or water-vapor-barrier layers because of its better water retention, mechanical strength, and WVTR performance, whereas BC/CMC-4 may be more suitable for absorbent or re-hydratable substrates requiring rapid water uptake. Nevertheless, further application-specific tests, such as oxygen permeability, antimicrobial activity, cytotoxicity, biocompatibility, and long-term stability, are still required before practical packaging- or biomedical-related applications can be confirmed.

## 5. Conclusions

In this study, a high-yield bacterial cellulose-producing strain, XNH6, screened from kombucha, was used to fabricate BC/CMC composite films with tunable structure and performance by introducing different concentrations of CMC during BC biosynthesis and subsequently applying citric acid post-treatment. The results showed that CMC effectively participated in the construction of the BC fibrillar network during biosynthesis. After citric acid treatment, FTIR and XPS analyses indicated changes in oxygen-containing functional groups and suggested possible ester-linkage-assisted stabilization within the composite network. However, these spectroscopic results were interpreted as qualitative evidence and were not used to quantify the degree of cross-linking. The composite structure preserved the cellulose I crystalline characteristics of BC while promoting fiber bundling, network densification, and pore optimization, thereby enabling the coordinated enhancement of microstructure and macroscopic properties.

Compared with neat BC, the BC/CMC composite films exhibited higher hydrophilicity, improved water-retention and re-hydration capacities, lower water vapor transmission rates, and enhanced rheological response and mechanical strength, indicating that in situ compositing combined with citric acid post-treatment helped generate a denser and more functional film structure. The effects of CMC concentration were clearly differentiated: BC/CMC-3 showed superior water retention, tensile performance, and water vapor barrier behavior, whereas BC/CMC-4 showed better re-hydration capacity and surface wettability.

Overall, this study showed that different CMC contents can effectively modulate the structure and properties of BC/CMC composite films, providing a basis for their potential use in moisture-management-related applications.

## Figures and Tables

**Table 1:** Preparation conditions of BC and BC/CMC composite films.

Sample	CMC Concentration in HS Medium	Culture Volume	Inoculum	Fermentation Condition	Citric Acid Treatment	Final Film
BC	0 g/L	20 mL	10% (*v*/*v*)	30 °C, 6 d, static	untreated	Pure BC film
BC/CMC-1	0.2 g/L	20 mL	10% (*v*/*v*)	30 °C, 6 d, static	10% CA, 140 °C, 15 min	BC/CMC composite film
BC/CMC-2	0.4 g/L	20 mL	10% (*v*/*v*)	30 °C, 6 d, static	10% CA, 140 °C, 15 min	BC/CMC composite film
BC/CMC-3	0.6 g/L	20 mL	10% (*v*/*v*)	30 °C, 6 d, static	10% CA, 140 °C, 15 min	BC/CMC composite film
BC/CMC-4	0.8 g/L	20 mL	10% (*v*/*v*)	30 °C, 6 d, static	10% CA, 140 °C, 15 min	BC/CMC composite film
BC/CMC-5	1.0 g/L	20 mL	10% (*v*/*v*)	30 °C, 6 d, static	10% CA, 140 °C, 15 min	BC/CMC composite film

**Table 2:** XPS surface elemental composition and C 1s deconvolution results of BC, BC/CMC-3, and BC/CMC-4 films.

Sample	C/at. %	O/at. %	O/C	C–C/C–H/%	C–O/C–O–C/%	O–C–O/C=O/%	O–C=O/%
BC	75.45	22.01	0.292	38.71	47.15	12.12	2
BC/CMC-3	63.59	32.63	0.513	23.3	57.18	16.73	2.79
BC/CMC-4	63.93	33.47	0.524	23.38	55.39	19.73	1.49

Note: The atomic percentages of C and O were obtained from the XPS survey spectra after sensitivity factor correction; the relative contents of the C 1s components were calculated based on the fitted peak areas. Only the major elements C and O are listed in the table, while minor elements are not discussed in detail.

**Table 3:** Crystallinity indices of BC/CMC composite films.

Sample	2θ (°)			Crystallinity (%)
	(101)	(110)	(200)	
BC	14.00	16.00	22.20	87.49
BC/CMC-1	14.72	16.61	22.85	81.59
BC/CMC-2	14.53	16.55	22.80	80.92
BC/CMC-3	14.62	16.72	22.76	77.69
BC/CMC-4	14.34	16.37	22.43	82.57
BC/CMC-5	14.58	16.57	22.66	80.67

## Data Availability

The original contributions presented in this study are included in the article. Further inquiries can be directed to the corresponding author.

## Abbreviations

FTIR	Fourier transform infrared spectroscopy
XPS	X-ray photoelectron spectrometer
XRD	X-Ray diffraction
SEM	Scanning electron microscope
HS	Hestrin-Schramm
